# Future theranostic strategies: emerging ovarian cancer biomarkers to bridge the gap between diagnosis and treatment

**DOI:** 10.3389/fddev.2024.1339936

**Published:** 2024-02-01

**Authors:** Weranga Rajapaksha, Riya Khetan, Ian R. D. Johnson, Anton Blencowe, Sanjay Garg, Hugo Albrecht, Todd A. Gillam

**Affiliations:** ^1^ Centre for Pharmaceutical Innovation (CPI), Clinical and Health Sciences, University of South Australia, Adelaide, SA, Australia; ^2^ Applied Chemistry and Translational Biomaterials (ACTB) Group, Clinical and Health Sciences, University of South Australia, Adelaide, SA, Australia

**Keywords:** ovarian cancer, tumor, biomarkers, therapeutics, diagnostics, theranostics, theragnostics, nanomedicine

## Abstract

Ovarian cancers are a complex and heterogenic group of malignancies that are difficult to detect, diagnose and treat. Fortunately, considerable knowledge of ovarian cancer specific biomarkers has been generated, that is pertinent to the development of novel theranostic platforms by combining therapies and diagnostics. Genomic and proteomic data has been invaluable in providing critical biomolecular targets for ovarian cancer theranostic approaches. Exploitation of the wealth of biomarker research that has been conducted offers viable targets as beacons for ovarian cancer detection, diagnosis, and therapeutic targeting. These markers can be used in theranostics, a treatment strategy that combines therapy and diagnostics and is common in nuclear medicine, where radionuclides are used for both diagnosis and treatment. The development of theranostics has taken substantial focus in recent years in the battle against ovarian cancer. Yet to date only one theranostic technology has emerged in clinical practice. However, given the wealth of ovarian cancer biomarkers the field is poised to see the emergence of revolutionary disease treatment and monitoring outcomes through their incorporation into the development of theranostic strategies. The future of ovarian cancer treatment is set to enable precise diagnosis, targeted treatment, and vigilant monitoring. This review aims to assess the status of ovarian cancer diagnostic tools and biomarkers in practice, clinical development, or pre-clinical development, highlighting newly emerging theranostic applications.

## 1 Introduction

Ovarian cancer comprises several subclasses of heterogeneous and aggressive malignancies that pose a significant health challenge to women worldwide (Eisenhauer et al., 2018). Ovarian cancer is the fifth most common cancer in women, and the second most common gynecological cancer (Arora et al., 2023). Globally, approximately 300,000 new cases and 185,000 deaths were attributed to ovarian cancer in 2020 (Inggriani et al., 2023) . In 2040, nearly 42% more women worldwide are predicted to be diagnosed with ovarian cancer, bringing the total number of new cases to over 445,000 (Zoń and Bednarek, 2023). Despite advances in cancer care, ovarian cancer remains a major contributor to cancer-related deaths among women (Ferlay et al., 2015; Cabasag et al., 2022).

The pathogenesis of ovarian cancer is complex with several subtypes (Zhao et al., 2023). Epithelial ovarian cancer (EOC) accounts for about 90% of cases (Sung et al., 2023) and is grouped into type I and type II grades based on distinct molecular and clinical characteristics (Grabowska-Derlatka et al., 2023). Type I EOCs comprise low-grade serous, mucinous ovarian carcinomas, ovarian endometrioid and ovarian clear cell carcinomas (Li et al., 2023) that are thought to develop from benign cysts, often associated with endometriosis (Hsieh et al., 2023; Saida et al., 2023). Type I EOCs are less aggressive than type II EOCs, usually presenting at an early stage of the disease pathogenesis (Jayson et al., 2014). Type II EOCs are more aggressive high-grade serous carcinomas and are often diagnosed in the late stage, and are responsible for the majority of ovarian cancer-related deaths (Stewart et al., 2019; Babaier and Ghatage, 2020; Grabowska-Derlatka et al., 2023).

Despite the distinct characteristics and aggressiveness of type I and type II EOCs, the diagnosis of ovarian cancer remains a significant challenge (Huang et al., 2023). The symptoms of ovarian cancer, include abdominal bloating, pelvic or abdominal pain, difficulty eating, and urinary urgency (Brain et al., 2014). These early symptoms are non-specific and can be overlooked by patients through misattribution to other pathologies. This often results in late medical examination, delaying both diagnosis and effective treatment (Rampes and Choy, 2022; Huang et al., 2023). Diagnosis requires a combination of imaging approaches, blood-based screens, and histopathological studies that require invasive biopsies or surgeries (Rubin et al., 2020; Sun et al., 2020; Tuncer et al., 2020). Traditional imaging methods, such as transabdominal and transvaginal sonography (Miao et al., 2023), computed tomography (CT) (Kryzhanivska et al., 2023), magnetic resonance imaging (MRI) (Zhang et al., 2017), positron emission tomography (PET) (Li et al., 2023), and color doppler imaging (Wang et al., 2023) are often not sensitive enough to detect ovarian cancer early or to accurately assess the extent of the disease (St Lorenz et al., 2023). Additionally, the technical limitations of these imaging techniques in assessing tumor characteristics and metastasis can lead to misdiagnosis or inaccurate staging (Suppiah, 2018). Furthermore, the high cost of these diagnostics and the absence of a well-defined detection point often lead to delays in diagnosis, further compromising treatment outcomes (Zhang et al., 2023). Hence there is an urgent need for the development of more effective diagnostic platforms and strategies.

Biomarkers, which are related to the altered cellular biology that underpins disease pathogenesis, have become integral to cancer therapeutics, aiding in various stages of a patient’s diagnostic and therapeutic process. Looking ahead, biomarkers are expected to be increasingly utilized in liquid biopsies and multiple samplings to delve deeper into tumor heterogeneity and identify drug resistance mechanisms (Louie et al., 2021). Further to this there is a push to couple these emerging diagnostic tools with therapeutic solutions to produce theranostic agents capable of assisting the detection, monitoring and treatment of ovarian cancers.

**FIGURE 1:**
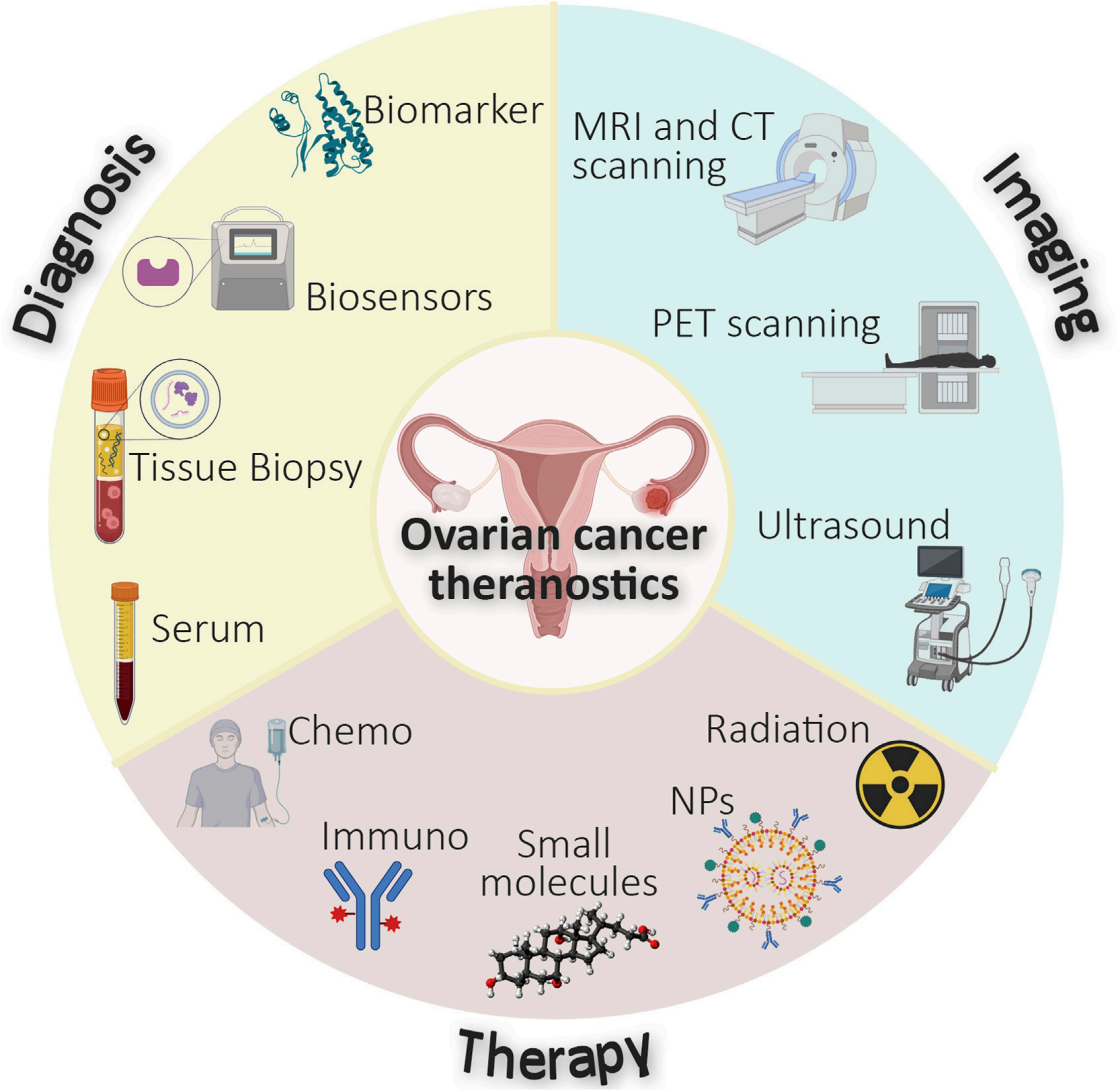
Schematic diagram representing the currently integrated diagnostic, imaging, and therapeutic approaches in ovarian cancer.

The conventional management of ovarian cancers involves a multimodal approach, utilizing surgery, chemotherapy, targeted therapies and in some cases, immunotherapy (Agyemang et al., 2022). As illustrated in Figure 1, treatment decisions are informed by diagnosis and imaging, integrating factors such as disease stage, histological subtype, patient’s age and overall health, and the presence of specific molecular markers (Morrison et al., 2012). Surgery is the cornerstone of ovarian cancer treatment and aims at removing as much tumor tissue as possible (Morrison et al., 2012), whilst chemotherapy and in some cases small molecule-based therapies (Figure 1) are commonly used as neoadjuvant and adjuvant treatment in conjunction with surgery (Kuroki and Guntupalli, 2020; Coleridge et al., 2021). Combination therapies, and the use of intraperitoneal chemotherapy, have shown improved survival rates in certain patient populations (Pasqual et al., 2023). Recently, immunotherapy (Figure 1) has become an emerging treatment option that enables immune cells to recognize and attack cancer cells (Ou et al., 2023). These therapies are designed to specifically target molecular alterations present in cancer cells.

Despite advancements in diagnostics, ovarian cancers remain difficult to treat, with a 5-year survival rate from advanced-stage disease of only 40% (Yang et al., 2023). Therefore, there is a need for new and more effective treatments that are tailored to the specific subclass of each patient’s tumor. Additionally, better methods to monitor recurrence are required. These needs can potentially be met by developing efficient personalized medicines and theranostic agents. Targeting ovarian cancer-specific molecular signatures could revolutionize ovarian cancer management by improving platforms for early detection and facilitating the monitoring of treatment responses (Khetan et al., 2022). Nanomedicines with high encapsulation capacity for therapeutic and diagnostic agents (Figure 1) have the potential to address the limited densities of specific molecular markers expressed on cancer cells and enable early diagnosis and effective treatment (Pardeshi et al., 2023). The amalgamation of both therapeutic and diagnostic elements within a single theranostic agent is increasingly attractive. Agents which feature these two properties have been established using various radioisotopes (Figure 1) and currently exist for a variety of cancers such as neuroblastoma ([^131^I]metaiodobenzylguanidine) and thyroid cancer (^123^I/^131^I). The theranostic capability to provide therapeutic benefit whilst reporting on disease status, such as tumor location and size (Maity et al., 2022) is paramount to the development of effective treatment strategies for ovarian cancer (amongst other diseases). This is exemplified by the incorporation of imaging modalities into targeted drug delivery systems, such as antibody-drug conjugates (ADCs), which contribute an ability to visualize the distribution of the ADCs within tumors (Minnix et al., 2020).

In the field of targeted cancer therapy, several challenges need to be addressed to enhance the potential of theranostics. The first challenge is the targeted delivery of theranostic agents with cytotoxic payloads (conventional chemotherapeutic agents) to tumor sites (Tsourkas, 2019). The second challenge is the reliance on the enhanced-permeability-retention (EPR) effect for the accumulation of systemically administered nanoparticle-based drug delivery systems within tumor tissue, which is driven by passive diffusion and facilitated by ‘leaky’ tumor vasculature (Amraee et al., 2023). The third challenge is the need for active targeting, utilizing high-affinity molecules like antibodies, antibody fragments, and peptides, to achieve targeted drug and theranostic delivery to ovarian cancer cells (Slastnikova et al., 2018; Todaro et al., 2023). This approach offers a high degree of cell-specific selectivity and a pathway for active cellular internalization. However, initial delivery often relies on the passive targeting of tumors *via* the EPR effect when using a nanoparticle system (Anani et al., 2021). The fourth challenge is the presence of additional biological barriers, such as the desmoplastic tumor microenvironment and increased interstitial pressure, which must be overcome to realize truly efficient passive tumor targeting using nanoparticle delivery systems (Jain, 2012). The fifth challenge is the need for optimization of nanoparticle size and stability (circulation time) to progress towards the development of successful treatment strategies (Nakamura et al., 2016). Lastly, the incorporation of motifs to target and trigger the active internalization of these nanoparticles is a promising approach to overcoming the “binding site barrier” and improve targeted drug delivery (Anani et al., 2021). However, this also presents a challenge that needs to be addressed.

Whilst still in the early stages of development, theranostics have the potential to revolutionize the diagnosis and treatment of ovarian cancer to address several unmet needs in ovarian cancer care. This review focuses on recent developments in emerging biomarkers for ovarian cancer theranostic development. By comprehensively analyzing studies, clinical trials, and pre-clinical research, we aim to highlight the feasibility of these biomarkers in bridging the gap between diagnosis and treatment, ultimately advancing ovarian cancer management, and improving treatment outcomes.

## 2 Ovarian cancer diagnosis

The diagnosis of ovarian cancer has seen significant progress, driven by the integration of biomarkers and advanced imaging techniques (Li and Wang, 2023). For instance, a combination of mucin 16 (MUC16 also known as carbohydrate antigen 125 or CA-125), with pelvic ultrasound achieved a higher specificity for ovarian cancer detection (Niu et al., 2023), and the synergy of MUC16 and human epididymis protein 4 (HE4) biomarker testing with transvaginal sonography has contributed to enhanced ovarian cancer screening (Stewart et al., 2019). These advancements have reformed our approach to diagnosing ovarian cancer, providing valuable insights into disease prognosis, and treatment strategies.

With the increasing demand for personalized treatment, the role of novel biomarkers has the potential to enhance the prediction and diagnostic approach towards disease control. Numerous studies have underscored the significance of biomarkers, such as protein molecules, nucleic acids, and genetic alterations, which are currently in practice, or in various stages of pre-clinical or clinical development.

### 2.1 Implementation of biomarkers in current clinical practice

There are a variety of biomarkers that are currently utilized in clinical practice to detect and predict different stages of ovarian cancer (summarized in Table 1). One of the most notable diagnostic biomarkers is MUC16 (Ferrer, 2023), which is widely used in ovarian cancer assessment, however, it has limited use as an independent endpoint for drug treatment selection (Herzog et al., 2017). MUC16’s specificity is hampered by its elevation in various non-cancerous conditions, such as endometriosis, cirrhosis, menstruation, pregnancy, pelvic inflammation, and uterine leiomyomata (Atallah et al., 2021). Due to these limitations, MUC16 as a standalone marker for ovarian cancer detection is not recommended (Liu et al., 2023). HE4 is another commonly used ovarian cancer biomarker with comparable sensitivity to MUC16 when detected in serum (Bilgi Kamaç et al., 2023; Washington et al., 2023). Moreover, HE4 is resistant to peritoneal irritation and therefore produces fewer false-positive findings when used to distinguish between malignant and benign pelvic masses (Lycke et al., 2021). Various combinations of biomarkers have shown promise in the development of more reliable ovarian cancer screening tools, which are also amenable to early ovarian cancer detection. Notably, OVA1^®^ is a United States (US) Food and Drug Administration (FDA) approved test, which includes apolipoprotein A-I (APOA1), transthyretin (TTR), transferrin (TF), β2-microglobulin (B2M), along with MUC16, and has demonstrated effectiveness in detecting early-stage ovarian cancer (Zamanian-Daryoush and DiDonato, 2015). Additionally, multiplexed magnetic nanoparticle-antibody conjugates combining MUC16, B2M, and APOA1 achieved high sensitivity (94%) and specificity (98%) in distinguishing early-stage ovarian cancer patients from healthy individuals (Pal et al., 2015). In addition, the combination of TTR and APOA1 with MUC16 and TF has shown a 96% overall sensitivity for early detection of ovarian cancer (Nosov et al., 2009).

**TABLE 1:** Biomarkers in clinical use.

Biomarker	Type	Source	Detected in	Application	References
APOA1 (ApoA-1)	Protein	Tissue	Plasma	Diagnosis, prognosis	Stavnes et al. (2014), Pal et al. (2015), Moore et al. (2019), Reilly et al. (2023)
B2M (β2-microglobulin)	Protein	Tissue	Serum	Diagnosis	Pal et al. (2015), Reilly et al. (2023)
FOLR1	Receptor	Tissue	Tissue biopsy	Diagnosis, therapy	Dilawari et al. (2023), Nwabufo (2023)
HE4 (Human epididymis protein 4)	Glycoprotein	Tissue	Serum	Prognosis, dual marker with MUC16	Barr et al. (2022), Samborski et al. (2022)
MUC16 (CA-125)	Glycoprotein	Tissue	Serum	Diagnosis, prognosis, disease stabilization, targeted therapy	Zhao et al. (2023b), Luo et al. (2023), Song et al. (2023)
TF (Transferrin)	Protein	Blood	Serum	Diagnosis	Ivanova et al. (2022), Reilly et al. (2023)
TTR (Transthyretin)	Protein	Tissue	Serum	Diagnosis	Clarke et al. (2011), Moore et al. (2019), Reilly et al. (2023)

Folate receptor alpha (FOLR1), which is a glycosylphosphatidylinositol-anchored glycoprotein has emerged as a promising biomarker for the detection of ovarian cancer, exhibiting notable potential and versatility in clinical applications (Leung et al., 2013; Bax et al., 2023). Serum analysis has revealed elevated levels of FOLR1 in patients with ovarian cancer compared to those with benign gynecological conditions and healthy controls (Zhang et al., 2022). FOLR1 demonstrates considerable diagnostic value, surpassing the performance of other serum biomarkers. As an epithelial cell surface receptor, FOLR1 or its components may be shed into the circulation, making it a viable candidate as a serum marker for ovarian cancer (Bax et al., 2023). One of the significant features of FOLR1 is its high binding affinity for folic acid and its derivatives (Gandidzanwa et al., 2023). In 2022, the US FDA granted accelerated approval for mirvetuximab soravtansine-gynx (MIRV) alongside the companion diagnostic device VENTANA FOLR1 (FOLR-2.1) RxDx for the treatment of adult patients with FOLR1-positive ovarian cancer (Dilawari et al., 2023). The use of folate-conjugated fluorescent dyes and radiolabels for tumor imaging is a groundbreaking advancement in ovarian cancer detection and treatment (Azaïs et al., 2016). These tools not only provide surgeons with real-time intraoperative imaging guidance, enhancing surgical precision, but also serve as versatile diagnostic tools, enabling more accurate and comprehensive tumor characterization (Hekman et al., 2017; Numasawa et al., 2020; García de Jalón et al., 2023). This dual functionality significantly improves the clinical management of ovarian cancer, paving the way for more personalized and effective treatment strategies.

Several traditional biomarkers have demonstrated significant potential in theranostics. For instance, TTR is associated with cancer and its presence in ascitic fluid (Gericke et al., 2005). It is a part of thyroxine-binding globulin and albumin, responsible for transporting thyroid hormones in the bloodstream and is involved in vitamin A metabolism. Another biomarker, APOA1 is a major component of high-density lipoprotein (Kim et al., 2012; Zamanian-Daryoush and DiDonato, 2015). Higher APOA1 mRNA levels in pre-chemotherapy effusions from advanced-stage ovarian cancer patients are observed to be an independent prognostic marker with longer overall survival (Tuft Stavnes et al., 2014). B2M is a small, non-glycosylated polypeptide (Hofbauer et al., 2021) elevated in ovarian cancer and critical to mediating tumorigenesis, metastasis, and angiogenesis through various signaling pathways (Sun et al., 2016). B2M elevation is often associated with increased cell proliferation, making B2M a valuable biomarker for ovarian cancer diagnosis. However, the clinical development of TTR, B2M and APOA1 as biomarkers for ovarian cancer is still ongoing.

### 2.2 Biomarkers in clinical development

Numerous biomarkers are currently under investigation for the targeted approach towards early diagnosis and prediction of different tumor stages of ovarian cancer (summarized in Table 2). Chitinase-3-like protein 1 (CHI3L1), also known as YKL-40, is suggested to have potential as a superior marker to MUC16 for the early diagnosis of EOC (Høgdall et al., 2009; Zou et al., 2010). CHI3L1 is involved in extracellular matrix degradation and promotion of angiogenesis through a vascular endothelial growth factor (VEGF)-independent pathway (Kotowicz et al., 2017). *In vitro* studies have revealed its association with VEGF upregulation and tumor angiogenesis, while animal studies have demonstrated that inhibiting CHI3L1 leads to reduced angiogenesis, tumor development, and metastasis (Shao, 2013; Kahramanoğlu et al., 2018). Recent research has revealed that both neutrophils and tumor cells can express and release CHI3L1 into the bloodstream, resulting in elevated serum levels in several cancer types (Zhao et al., 2020). Kahramanoğlu et al. (2018) suggest that preoperative serum levels of CHI3L1 in patients with serous EOC were significantly higher than those with benign ovarian tumors. This finding suggests that CHI3L1 may serve as a better predictor of ovarian cancer compared to MUC16, with moderate-to-high sensitivity (80%) and specificity (70%) when a specific cutoff level is applied (Kahramanoğlu et al., 2018; Deveci et al., 2019).

**TABLE 2:** Biomarkers in clinical development.

Biomarker	Type	Source	Detected in	Application	Clinical trial number	References
CD24	GPI-anchored glycosylated protein	Tissue	Tissue biopsy	Diagnosis, targeted therapy	NA	Wang et al. (2015), Soltész et al. (2019), Ashihara et al. (2020), Nagare et al. (2020)
CHI3L1 (YKL40)	Protein	Tissue	Serum	Diagnosis, targeted therapy	NCT00899093, NCT05810701	Kahramanoğlu et al. (2018), Deveci et al. (2019), Chang et al. (2022)
FLT4 (VEGFR3)	Tyrosine kinase receptors	Tissue	Tissue biopsy	Diagnosis, targeted therapy	NCT05494580	Klasa-Mazurkiewicz et al. (2011), Babaei et al. (2023b)
KLK6, KLK10	Serine proteases	Blood	Serum	Diagnosis	NA	Pépin et al. (2011), Koh et al. (2012), Geng et al. (2017)
MSLN	GPI-anchored Glycoprotein	Tissue	Serum	Diagnosis, targeted therapy	NCT00155740	Yildiz et al. (2019), Tegeler et al. (2022), Weidemann et al. (2023)
Nectin-2	Glycoprotein	Tissue	Serum, tissue	Diagnosis	NCT03667716	Bekes et al. (2019), Zeng et al. (2021), Sim et al. (2022)
Nectin-4	Glycoprotein	Tissue	Serum, tissue	Diagnosis, targeted therapy	NCT02091999, NCT04561362	Rogmans et al. (2022)
PSN (Prostasin)	GPI-anchored extracellular serine protease	Seminal fluid	Serum	Diagnosis	NA	Tamir et al. (2016), Bastani et al. (2017)
SLC34A2 (NaPi2B)	Solute carrier phosphate transport protein	Tissue	Tissue biopsy	Diagnosis, targeted therapy	NCT03319628	Lin et al. (2015), Banerjee et al. (2018), Nurgalieva et al. (2021), Banerjee et al. (2023b)

Vascular endothelial growth factor receptors (VEGFRs) are a family of transmembrane tyrosine kinase receptors involved in signal transduction pathways to control vascular development of ovarian cancer (Babaei et al., 2023a). VEGFR3 is overexpressed in ovarian cancer cell lines promoting cell growth (Babaei et al., 2023b) and has been identified as a biomarker with potential for the diagnosis and prognosis of ovarian cancer (Klasa-Mazurkiewicz et al., 2011). Moreover, VEGF is a key mediator of angiogenesis and plays a significant role in ovarian cancer development by promoting the recruitment and proliferation of endothelial cells within the tumor (Liang et al., 2015). Elevated levels of VEGFA are associated with increased formation of new blood vessels in tumor tissue, and have become an important factor contributing towards improved accuracy of ovarian cancer diagnosis (Liang et al., 2015).

Mesothelin (MSLN) is a membrane-bound surface glycoprotein that has emerged as a promising candidate for ovarian cancer diagnosis (Rump et al., 2004). Unfortunately, its use is limited as a standalone target with a sensitivity of only 68%, raising concerns about its reliability for early detection (Madeira et al., 2016). Despite its limitations as a standalone marker, MSLN’s potential should not be overlooked since it could significantly enhance diagnostic accuracy if used in conjunction with other markers. Moreover, its potential for targeted theranostic applications could open new avenues for personalized treatment strategies in ovarian cancer patients.

Glycosylphosphatidylinositol (GPI) anchored prostasin (PSN) is a promising marker for ovarian cancer diagnosis with sensitivity and specificity as high as 92% and 94%, respectively. PSN is a trypsin-like proteinase known as a channel-activating protease that is linked to sodium regulation and inhibits cancer cell proliferation and invasion (Tamir et al., 2016). PSN was first detected within the prostate (Tamir et al., 2016), and found to be overexpressed in various EOCs (Ma et al., 2014; xiang et al., 2013). Thus, PSN could serve as a potential early detection biomarker independently of MUC16.

Cell adhesion molecules of the nectin family (Rogmans et al., 2022), and the F‐actin‐binding protein afadin (Hiremath et al., 2023), form homophilic and heterophilic trans‐dimers which present as useful biomarkers for diagnostic, therapeutic and potentially theranostic applications (Bekes et al., 2019). Nectins play a vital role in tumor development, mediating tumorigenesis, metastasis, and angiogenesis through various signaling pathways (Boylan et al., 2016). Among the nectins, nectin‐2, also known as CD112 coordinates various cellular functions critical for survival, proliferation, adhesion, migration, and differentiation (Bekes et al., 2019). The presence of nectin‐4 as a blood-based marker for ovarian cancer cells implies that it is involved in regulating endothelial functions, such as migration, proliferation, and invasion (Rogmans et al., 2022).

CD24 is being studied as a candidate for the future development of targeted therapeutics (Panagiotou et al., 2022). This GPI-anchored cell adhesion protein (Tarhriz et al., 2019) is known to be overexpressed in ovarian cancer (Li et al., 2023), while barely detected in healthy tissues (Yang et al., 2023). CD24 is notably and almost universally expressed in EOC, with prevalence ranging from 70 to 100% (Nakamura et al., 2017; Kleinmanns et al., 2020). This expression pattern highlights its potential as a significant marker in the context of EOC diagnosis and targeted therapeutic strategies (Kleinmanns et al., 2020).

Similarly, sodium-dependent phosphate transporter NaPi2b also known as SLC34A2, a sodium-dependent phosphate transporter, is overexpressed in high-grade serous ovarian carcinoma (HGSOC) (Banerjee et al., 2023a). This could be promising for ovarian cancer detection and could facilitate the identification of patients who are most likely to benefit from specific targeted therapies. SLC34A2 is also regulated by PAX8, a master transcription factor associated with ovarian cancer cell survival and the development of female reproductive systems, suggesting that SLC34A2 may play an important role in tumorigenesis (Bondeson et al., 2022). SLC34A2 typically has stable expression throughout ovarian cancer disease pathogenesis and treatment, as evidenced by the consistent SLC34A2 expression observed in longitudinal tissue samples (Banerjee et al., 2023b). Utilizing SLC34A2 as a biomarker in ovarian cancer could help to address the challenges associated with early stage ovarian cancer diagnosis, which could drastically improve patient outcomes.

Lastly, human tissue kallikreins (KLKs), a family of 15 members, are expressed in various tissues including the breast, ovary, prostate, and testis (Koh et al., 2011). KLK6 and KLK10 in particular are highly expressed in ovarian cancer (Koh et al., 2011). Whilst the expression of KLKs is not specific enough for detecting disseminated disease, KLK expression aids in distinguishing ovarian cancer from other malignancies or non-malignant conditions (Oikonomopoulou et al., 2006).

### 2.3 Biomarkers in pre-clinical development

Newly emerging biomarkers for ovarian cancer (summarized in Table 3) include epithelial cell adhesion molecule (EPCAM), syndecans, R-spondin, and several G protein-coupled receptors (GPCRs), including the estrogen receptor GPER1, that are currently being investigated in pre-clinical studies. EPCAM, also known as CD326, is expressed in normal human epithelial cells and most epithelial tumor cells (Ding et al., 2023). EPCAM plays a crucial role in promoting cell cycle, tumor cell proliferation, migration and immune evasion in various epithelial cancers (Li et al., 2023). In ovarian cancer, EPCAM is often highly expressed in malignant tumors, correlating with poor prognosis (Tayama et al., 2017). Patients with high EPCAM expression are more likely to be chemo-resistant and suffer poor survival rates (Ibrahim et al., 2023). This makes EPCAM a promising biomarker for predicting response to chemotherapy in ovarian cancer.

**TABLE 3:** Biomarkers in pre-clinical development.

Biomarker	Type	Source	Detected in	Application	References
CXCL12	Chemokine protein	Tissue	Tissue biopsy	Diagnosis, targeted therapy	Salomonnson et al. (2013), Guo et al. (2014), Mao et al. (2017)
CXCR4	Protein	Tissue	Tissue biopsy	Diagnosis, prognosis, targeted therapy	Salomonnson et al. (2013), Guo et al. (2014), Li et al. (2014), Liu et al. (2014), Lim et al. (2023)
EPCAM	Transmembrane glycoprotein	Tissue	Tissue biopsy	Diagnosis, targeted therapy	Ploeg et al. (2023), van den Brand et al. (2020), Wang et al. (2022), Zheng et al. (2017)
FSHR	Transmembrane receptor	Tissue	Tissue biopsy	Diagnosis, prognosis	Deuster et al. (2019)
GPER1	Protein	Tissue	Tissue biopsy	Diagnosis	Long and Duan (2022)
LGR5/6	Protein	Tissue	Tissue biopsy	Diagnosis, prognosis	Yu et al. (2019), LEE et al. (2020), Kim et al. (2022)
LHCGR	Transmembrane receptor	Tissue	Tissue biopsy	Diagnosis, prognosis, therapy	Kawai et al. (2018), Zhong et al. (2019)
LPAR2/3	Protein	Tissue	Tissue biopsy		Xiong et al. (2016), Xiong et al. (2017)
LPA	Bioactive phospholipid	Tissue	Serum	Diagnosis	Ahmadi et al. (2023), Tarannum et al. (2023)
RSPO1	Secreted protein	Tissue	Serum, tissue biopsy	Diagnosis, targeted therapy	Liu et al. (2019), LEE et al. (2020)
SDC3	Transmembrane proteins	Tissue	Serum, tissue biopsy	Diagnosis, targeted therapy	Hillemeyer et al. (2022)

Syndecans are integral transmembrane heparan sulfate proteoglycans involved in organizing cellular signaling processes at the cell surface (Hillemeyer et al., 2022). Syndecans interact with cytokines, signaling receptors, proteases, and components of the extracellular matrix, to control cellular processes such as proliferation, metastasis, angiogenesis, and inflammation (Espinoza-Sánchez and Götte, 2020). The upregulation of syndecan-3 (SDC3), has been demonstrated in various gene expression datasets, highlighting their utility in identifying the presence of ovarian cancer and even distinguishing metastatic lesions. Detecting SDC3 in ovarian cancer tissues can enable more accurate and early detection of the disease. (Kulbe et al., 2019; Guo et al., 2022; Hillemeyer et al., 2022).

Several GPCRs are overexpressed in ovarian cancer including the lysophosphatidic acid receptor (LPAR), C-X-C chemokine receptor type 4 (CXCR4), follicle-stimulating hormone receptor (FSHR) and luteinizing hormone/choriogonadotropin receptor (LHCGR) (Khetan et al., 2022). Lysophosphatidic acid (LPA) was identified as a major regulatory factor stimulating the expression of numerous genes associated with angiogenesis and metastasis of ovarian cancer (Pua et al., 2009). LPA is detected at very high concentrations in ascitic fluid and can serve as a diagnostic marker to assess disease progression and metastasis (Yu et al., 2016). LPARs are overexpressed in ovarian cancer cells and tissues, specifically, LPAR2 and LPAR3 (Khetan et al., 2022). Chemokines regulate many cellular processes, including cell migration, proliferation, and differentiation. Ovarian epithelial carcinoma specifically express the chemotactic factor C-X-C motif chemokine ligand 12 (CXCL12) and its receptor CXCR4, which have been identified as potential prognostic markers (Lim et al., 2023).

Similarly, LGR5 and LGR6 are two members of the leucine rich repeat (LGR) containing GPCRs family and are expressed on the surface of ovarian cancer tissues (Schindler et al., 2017). The high expression of LGR5 and LGR6 mRNA in HGSOCs allows them to be constantly stimulated by WNT signaling, which leads to uncontrolled cell growth and tumor development (Wong et al., 2023). R-spondins (RSPOs) are cysteine-rich secreted glycoproteins that amplify and modulate WNT signals. RSPOs are the natural ligands that bind specifically to LGR5 and LGR6, activating the WNT signaling pathway (Wong et al., 2023). Among the RSPO family, RSPO1 is overexpressed in ovarian cancer tissues and has potential as a biomarker for diagnosis and targeted therapy (Liu et al., 2019; LEE et al., 2020).

G protein-coupled estrogen receptor 1 (GPER1 also known as GPR30) is overexpressed in a variety of tissues, including the ovary, breast, and endometrium (Smith et al., 2009). GPER1 is involved in cell growth, proliferation, and differentiation, and has been identified as a biomarker for ovarian cancer diagnosis and prognosis, and a potential target for therapeutic intervention (Liu et al., 2010). Similarly, FSHR and LHCGR are predominantly found in the ovary and uterus. FSHR interacts with the follicle-stimulating hormone and is responsible for the upregulation of oncogenic pathways and increased proliferation of EOC (Bordoloi et al., 2022) whereas LHCGR interacts with luteinizing hormone and gonadotrophins, and is necessary for follicular maturation and ovulation (Kumar and Idicula-Thomas, 2023). Cheung et al. reported that higher FSHR and LHCGR expression is associated with early stage, low grade ovarian cancer and the expression is reduced in HGSOC compared to benign ovarian tumors (Cheung et al., 2020). This would indicate that these GPCRs have great potential in playing the role of biomarkers for the detection and diagnosis of ovarian cancer at several tumor stages.

## 3 Current trends of novel and emerging ovarian cancer theranostic targets

**FIGURE 2:**
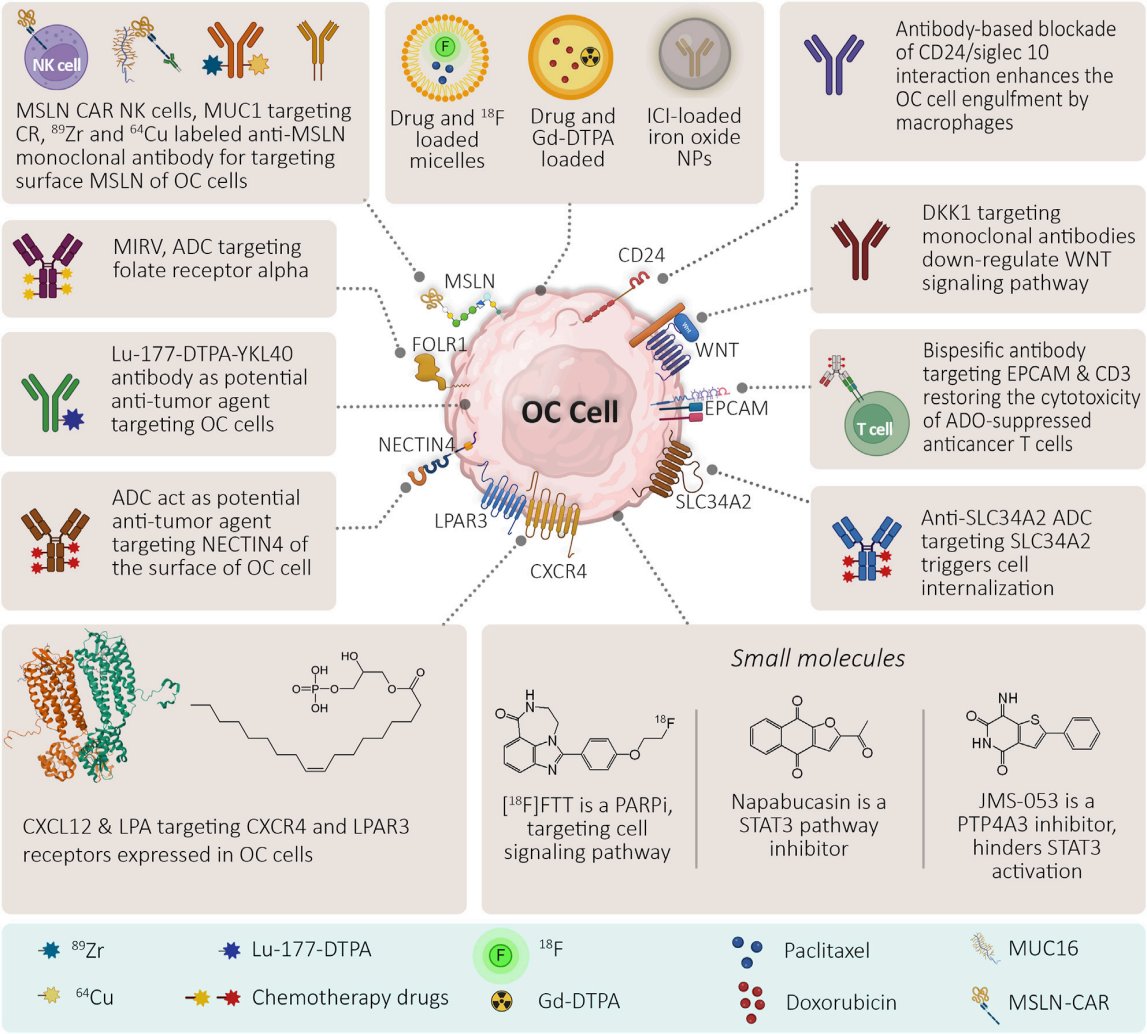
Emerging theranostic approaches in ovarian cancer.

Theranostics offer several advantages over conventional diagnostic and therapeutic approaches, such as improved accuracy, specificity, sensitivity, efficacy, safety, and personalization. This section explores some emerging biomarkers, nanomaterials and small molecules, and their prospects for the development of theranostic agents that can be used for the treatment of ovarian cancer (Figure 2).

### 3.1 MUC16 and MSLN as a novel theranostic target combination for future ovarian cancer treatment

Abnormal levels of MUC16 expression have been observed in 99% of ovarian cancer serous carcinomas (Morales-Vásquez et al., 2016). Binding of MUC16 to MSLN is known to activate the PI3K/AKT, ERK1/2, and JNK pathways to promote cell survival, migration, and invasion (Tang et al., 2013; Faust et al., 2022). An anti-MSLN antibody has been established to reverse these effects, leading to cell apoptosis (Klampatsa et al., 2021). Like MUC16, MSLN is over-expressed in ovarian cancer with limited expression in normal tissues (Weidemann et al., 2023). A soluble proteolytic fragment of MUC16 was recently shown to specifically bind to MSLN in an N-linked glycan-dependent mode (Huo et al., 2021). This interaction promotes cell migration through a mechanism that reduces the expression of dickkopf-1 (DKK1) whilst activating the SGK3/FOXO3 pathway. Most importantly, a monoclonal anti-MSLN antibody has been successfully applied to suppress tumor growth in a murine ovarian cancer xenograft model (Huo et al., 2021). MSLN has been further validated as a potential ovarian cancer target through the engineering of several recombinant immunotoxins. For example, a chimeric protein (SS1P) was composed of a MSLN-targeted variable antibody domain (Fv) fused to a fragment of *Pseudomonas* exotoxin A (PEA) (Liu et al., 2020), and several clinical trials have been carried out in combination with chemotherapeutic agents (NCT04503980, NCT05963100, NCT04562298, NCT06051695, NCT03692637, NCT01583686) (Pastan and Hassan, 2014; Santin et al., 2023; Shanghai Cell Therapy Group Co.,Ltd, 2020; Shen, 2023; Shanghai East Hospital, 2022; A2 Biotherapeutics Inc, 2023; Allife Medical Science and Technology Co. and Ltd, 2019; Rosenberg, 2019). LMB-100 is a variant of SS1P using a humanized fragment antigen-binding (Fab) region and a 24 kD PEA fragment instead of the previous 38 kD fragment (the 24 kD variant lacks a B-cell epitope, evading a non-desired immune response) (Liu et al., 2022). Finally, MSLN-targeting antibodies have been labeled with ^89^Zr (Prantner et al., 2015; Lamberts et al., 2016) and ^64^Cu (Kobayashi et al., 2015) demonstrating significant accumulation in MSLN-expressing tumors, paving the path towards imaging and therapeutic applications.

The MUC16/MSLN pair is emerging as a promising target for future cell-based theranostics. A very recent approach, based on the artificial expression of an engineered receptor in T-cells, used an extracellular MSLN 296–359 amino acid fragment (MSLN_296-359_) fused to an intracellular 4-1BB immune checkpoint molecule and a CD3ζ signaling fragment *via* a transmembrane sequence (Zhao et al., 2023). The intracellular 4-1BB and CD3ζ protein domains were shown to be activated in T-cells when MSLN_296-359_ bound successfully to MUC16 on ovarian cancer cells. This approach was further combined with a chimeric antigen receptor T (CAR-T) cell to artificially co-express the same chimeric receptor but with an extracellular MUC16-targeting single-chain variable fragment (scFv), instead of the MSLN_296-359_ fragment. Simultaneous expression of both receptor constructs was shown to synergistically drive T-cell mediated ovarian cancer cell killing (Zhao et al., 2023). Given the high specificity and selectivity of MUC16 and MSLN as diagnostic markers, they have very high potential as theranostic targets to diagnose, treat, and monitor ovarian cancers (Zhao et al., 2023).

### 3.2 FOLR1 is currently the only fully validated theranostic ovarian cancer target

MIRV is the first FDA-approved ADC targeting FOLR1 for the treatment of platinum-resistant ovarian cancer (Matulonis et al., 2023). In combination with the immunohistochemistry-based VENTANA FOLR1 (FOLR-2.1) RxDx assay, eligible patients for FOLR1 targeted treatment can be identified, leading to better stratified and more effective treatment. Numerous clinical trials with anti-FOLR1 antibodies are currently ongoing (NCT05870748, NCT05001282, NCT04274426, NCT05887609) (Banerjee et al., 2022; AGO Research GmbH, 2023; Elucida Oncology, 2023; ImmunoGen and Inc, 2023; Sutro Biopharma and Inc, 2023; Sutro Biopharma and Inc, 2023; University of Colorado and Denver, 2023). These includes farletuzumab, which demonstrated anti-tumor activities with significant improvements compared to standard chemotherapy (Herzog et al., 2023). The ADC MORAb-202, combining farletuzumab with the small molecule microtubule inhibitor Eribulin, has also demonstrated anti-cancer efficacy (Sakai et al., 2021). Additionally, other strategies like CAR-cytokine-induced killer (CIK) cell therapy targeting FOLR1 have shown promising results in pre-clinical studies (Zuo et al., 2017). CAR-CIK cells are engineered to recognize and attack tumor cells that express FOLR1 and effectively kill ovarian cancer cells *in vitro* and *in vivo*. Moreover, CAR-CIK cells were more potent than CAR-T cells in killing ovarian cancer cells (Song et al., 2016). TNB-928B is a novel T-cell engager with enhanced safety and specificity for the treatment of ovarian cancer. It has a bivalent binding arm for FOLR1, which allows it to selectively target FOLR1-overexpressing tumor cells. TNB-928B has been shown to induce preferential effector T-cell activation, proliferation, and selective cytotoxic activity on high FOLR1 expressing cells (Avanzino et al., 2022).

### 3.3 Potential of miscellaneous biomarker candidates to advance from clinical development to theranostic applications

CHI3L1 is implicated in promoting ovarian cancer cell proliferation, invasion, migration, tumor angiogenesis, chemoresistance, and has significant potential as a theranostic target (Shao et al., 2009). While CHI3L1 antibodies have high affinity for EOC cells, they do not directly induce apoptosis *in vivo* as a stand-alone (Chang et al., 2022). However, conjugating the antibodies with a therapeutic radionuclide Lu-177 complex has been shown to enhance its stability and anti-tumor efficacy in a mouse xenograft model. Monitoring of therapeutic responses has been demonstrated with nanoSPECT/CT^®^ using a diethylene triaminepentaacetic acid (DTPA) chelating agent.

The nectin‐afadin system, recently recognized for its role in modulating adherens junctions and tight junctions (Samanta and Almo, 2015), presents a promising avenue for a theranostic approach. VEGF stimulates nectin-2 expression leading to indirect regulation of endothelial cells (Bekes et al., 2019). A chimeric anti-nectin-2 antibody (c12G1) has been conjugated to a cytotoxic drug to demonstrate significant anti-tumor activity in ovarian cancer models (Sim et al., 2022). Another family member, nectin-4, is overexpressed in ovarian cancer tissue (Nabih et al., 2014) and plays a role in tumor cell proliferation, motility, and invasion. It also promotes angiogenesis and lymphangiogenesis, which are essential steps for cancer metastasis (Bekos et al., 2019). Nectin-4 had a higher sensitivity and specificity compared to the MUC16 standard biomarker, especially for early-stage ovarian cancer (Rogmans et al., 2022). Several strategies have been developed to target and inhibit nectin-4 activity. Enfortumab vedotin, an FDA approved ADC for the treatment of urothelial cancer (Tomiyama et al., 2020) has also shown promise in the treatment of other solid tumors, such as melanoma and breast cancer (Rosenberg et al., 2019; Shao et al., 2022). Nectin-4 may also be a promising target for imaging diagnostics and targeted radionuclide therapy. However, more research is needed to validate the feasibility and efficacy of nectin-4 targeting in the theranostic field. One such strategy is to use ADCs, which bind to nectin-4 on the surface of cancer cells and deliver a cytotoxicity payload directly to the cells. Another approach to targeting nectin-4 is to use CAR-T cells, expressing engineered nectin-4-targeting chimeric receptors. The CAR-T cells are then infused back into the patient, where they can attack and kill cancer cells that express nectin-4 (Bouleftour et al., 2022).

Immune checkpoints are regulatory molecules that modulate immune responses, primarily acting to limit excessive immune activity and maintain self-tolerance (Brom et al., 2022). Immune checkpoint inhibitors (ICIs) are small-molecule agents designed to counteract the inhibitory signals of these checkpoints. By binding to immune checkpoint molecules, ICIs can improve immune suppression, thereby enhancing the body’s natural anti-tumor immune responses (Tan et al., 2022). CD24 functions as an immune checkpoint in ovarian cancer (Gu et al., 2023), where cells evade phagocytosis by macrophages through the interaction of CD24 molecules with the inhibitory receptor sialic acid-binding immunoglobulin-like lectin 10 (Siglec-10) on tumor-associated macrophages. This interaction blocks the macrophage’s ability to engulf cancer cells (Barkal et al., 2019). However, antibody-based blockade of CD24 or Siglec-10 enhance cancer cell engulfment by macrophages, reducing tumor growth. The role of the CD24-Siglec-10 axis on suppression of anti-tumor immunity highlights its potential as a theranostic target in cancer treatment (Barkal et al., 2019).

Emerging evidence suggests that patients with high SLC34A2 levels at diagnosis tend to maintain high expression throughout their disease, emphasizing the value of timely biomarker testing (Banerjee et al., 2023b). SLC34A2 levels can be assessed using immunohistochemistry-based diagnostic assays; whereby patients with high expression can be directed toward anti-SLC34A2 ADC therapy (Kiyamova et al., 2011; Nurgalieva et al., 2021). Such ADCs are monoclonal antibodies directed against SLC34A2, linked to cytotoxic payloads. Upon binding to the tumor antigen, these ADCs are internalized to release their cytotoxic payload with high specificity into tumor cells expressing SLC34A2. This selectivity diminishes off-target toxicity which is a drawback to many conventional chemotherapies.

### 3.4 Assessment of experimental biomarkers as future theranostic targets

Several bispecific antibodies such as Catumaxomab, MT110 and M701 have been designed to simultaneously bind EPCAM and CD3, to induce a specific T-cell-mediated immune response against ovarian cancer cells (Li et al., 2023). Another bispecific antibody (CD73xEPCAM) binds to ovarian cancer cell surfaces in an EPCAM-directed manner and indirectly triggers anticancer T-cell activity in the tumor vicinity. The immune-boosting mechanism is mediated through selective antibody binding to CD73, thereby inhibiting its enzymatic activity, and preventing the conversion of adenosine monophosphate into immunosuppressive adenosine. CD73xEPCAM creates an immune suppressed environment around the tumor, facilitating cancer cell eradication (Ploeg et al., 2023). These findings highlight a promising strategy to use the CD73-adenosine immune checkpoint to prevent ovarian cancer cells from evading the immune system.

CXCR4 is a key contributor to cancer cell proliferation, migration, and invasion (Shi et al., 2020). LPA has been shown to enhance the expression of CXCR4 and its cognate ligand CXCL12 (Wang et al., 2014). Moreover, many cancer tissues also express high levels of LPA receptors (LPARs), most prominently LPAR2 and 3 (Bhattacharjee et al., 2023). The combination of LPAR and CXCR4-targeted therapies could also benefit from the development of a theranostic approach. Identifying patients with elevated LPA/LPAR levels and overactive CXCR4-CXCL12 signaling could provide a basis for the establishment of personalized treatments and disease monitoring strategies (Wang et al., 2014). The utilization of LPAR and CXCR4 in ovarian cancer showcases a favorable approach in both diagnosis and targeted therapy (Lim et al., 2023). Targeted interventions can disrupt LPAR and CXCR4 mediated systems and inhibit tumor growth and metastasis (Wang et al., 2014). This personalized approach involves administering therapies that specifically target the LPA-CXCR4 axis, potentially improving treatment efficacy and patient outcomes (Geraldo et al., 2021).

WNT signaling is a complex pathway that regulates many important cellular processes, including stem cell function, cell fate determination, tumorigenesis, and tumor progression (LEE et al., 2020). Various secreted WNT antagonists, such as the Cerberus protein, WNT inhibitory factor 1, secreted frizzled-related protein (SFRP), and DKK families, help regulate this pathway (Shizhuo et al., 2009). LGRs interact with RSPOs, which modulates the activity of ubiquitin ligases RNF43 and ZNRF3, enhancing WNT signaling in HGSOC (LEE et al., 2020). HGSOC exhibits an elevated expression of LGR5, LGR6, and RSPO1, pointing towards the RSPO1/LGR6 axis as a potential driver of increased WNT signaling. This axis may lead to uncovering novel therapeutic targets in the fight against ovarian cancer (Liu et al., 2019; LEE et al., 2020; Wong et al., 2023). Recent findings have revealed overexpression of DKK1 in ovarian cancer and inhibition of WNT signaling (Wei et al., 2020). However, DKK1 inhibition may not affect tumor growth in all ovarian cancer cases, but its overexpression alters anti-tumor immune populations within the tumor microenvironment, suggesting that it may be a new therapeutic target in EOC, especially when used in combination with immune-modulatory therapy (Betella et al., 2020). Developing theranostic approaches that capitalize on DKK1 and RSPO1 as therapeutic targets may pave the way for personalized treatment strategies tailored to the unique molecular profiles of ovarian cancer patients (Wang and Zhang, 2011; Klotz et al., 2022).

Signal transducer and activator of transcription 3 (STAT3) is another important transcription factor that regulates proliferation, survival, metastasis and invasion of ovarian cancer (Seo et al., 2023). From a diagnostic perspective, molecular profiling techniques can be employed to assess the activation status of the STAT3 pathway in ovarian cancer patients. This profiling includes the analysis of activated forms of STAT3 (Y^705^ phospho-STAT3) and the expression levels of downstream targets associated with cancer cell proliferation and survival. These molecular markers serve as diagnostic indicators, helping identify patients with persistent STAT3 activation who are suitable candidates for targeted therapy (Standing et al., 2023). From a therapeutic perspective, the novel small molecule LLL12B has emerged as a potent inhibitor of the STAT3 pathway in human ovarian cancer cells. LLL12B effectively suppresses STAT3 phosphorylation and downregulates the expression of downstream targets (Zhang et al., 2021). Beyond that, Napabucasin a STAT3 inhibitor, has demonstrated antitumor activity by inducing cell cycle arrest which triggers autophagy. This holds the potential to terminate cancer cell proliferation and survival (Liu et al., 2021).

The protein tyrosine phosphatase type IVA member 3 (PTP4A3 or PRL-3), is a dual-specificity phosphatase, with elevated expression in ovarian cancer (Mayinuer et al., 2013). PTP4A3 plays a critical role in dephosphorylating signaling molecules, such as SHP-2 phosphatase and p38 kinase (Lazo et al., 2023). Specifically, it is involved in a feed-forward loop with STAT3 (Lazo et al., 2023). The PTP4A phosphatase inhibitor, JMS-053 treatment reduces Y^705^ phospho-STAT3 in ovarian cancer cells. Inhibiting PTP4A3 with JMS-053 can potentially hinder the activation of STAT3. Furthermore, JMS-053 treatment rapidly increases the phosphorylation status of SHP-2 phosphatase and p38 kinase signaling which leads to cancer cell death. This highlights the potential therapeutic relevance of targeting PTP4A3 for ovarian cancer treatment. Monitoring PTP4A3 expression levels and STAT3 phosphorylation status in tumor tissues could be implemented to indicate personalized phosphatase inhibitor treatment for ovarian cancer (Lazo et al., 2023).

## 4 Nanoparticles: a platform for the incorporation of diagnostic and therapeutic elements

Nanomedicine has become a prominent solution to many cancer drug delivery strategies (Bhardwaj et al., 2023; Huang et al., 2023; Zhang et al., 2023). The use of specific ligands and responsive functionalities on nano-systems allows them to engage with targets and to release payloads at intended locations (Ray et al., 2017). Therapeutic nanocarriers can be comprised of liposomes, micelles, dendrimers, hydrogels, quantum dots, carbon-based nanocarriers (such as carbon nanotubes and bucky balls), and inorganic nanoparticles (i.e., silica, gold, iron oxide, and titanium dioxide based particles) (Alshawwa et al., 2022). These nanocarriers can also function as sensors, including magneto-resistive, electrical, and electrochemical sensors (Yang et al., 2022). Compared to traditional antibody-tracer conjugates, nanomaterial-based biosensors can offer superior sensitivity and selectivity due to their large surface area to volume, high conductivity, high electrocatalytic activity, and fast electron transfer rate (Eissa et al., 2022) that allow for more efficient and accurate detection of biological events, and enhanced sensitivity and selectivity, addressing the limitations of existing diagnostic methods (Foroozandeh et al., 2023; Rajaie et al., 2023).

Diagnostic approaches utilizing nano-sensors are presently being developed with responsivity to specific ovarian cancer biomarkers (Büyüktiryaki et al., 2017). Specific recognition can be achieved through the conjugation of aptamers or antibodies on the nanoparticle surface (Dhas et al., 2023). Responsive imaging agents can change properties upon interacting with cancer cells or responding to treatment (Sharma et al., 2017; Song et al., 2022). Nanoparticles can be developed to be suitable for multimodal imaging, which allows for the combination of imaging modalities, such as MRI, positron emission tomography (PET), and fluorescence imaging. Doing so can provide comprehensive real-time monitoring and information about tumor location, size, and response to treatment (Dougherty et al., 2015; Williams et al., 2018).

Nanotechnology based systems can be modified with additional combinatorial modalities such as drug encapsulation (Suardi et al., 2020), stimuli-responsive moieties (Guo et al., 2020; Zhang et al., 2020), chemical conjugation (Taheri-Ledari et al., 2022), surface tethered prodrugs (Song et al., 2019), and tracer agents (Asgari et al., 2021). Thus, nano-systems offer significant scope for the development of theranostics (Xue et al., 2021; Kashyap et al., 2023). Targeted drug delivery systems are useful vehicles for combining modalities to develop theranostic platforms, such as incorporating fluorescent dyes or magnetic nanoparticles into nanocarriers. Ideally these nanocarriers would be engineered to specifically target ovarian cancer cells or biomarkers by furnishing them with ligands that bind to overexpressed ovarian cancer receptors, ensuring selective drug delivery (Chen et al., 2014). For instance, redox-sensitive polymeric micelles containing paclitaxel have been used in the treatment of chemotherapy-resistant ovarian cancer (Mutlu-Agardan et al., 2020). These micelles have the potential for further refinement, enabling the incorporation of imaging probes, thus serving a dual role in both the diagnosis and treatment of ovarian cancer (Han et al., 2019; Zhang et al., 2023). Additionally, their visibility may be enhanced through the introduction of a radioactive isotope like fluorine-18 (^18^F), which could facilitate their tracking *via* PET imaging (Francis and Wuest, 2021). This capability may be instrumental in verifying their precise journey within the body, ensuring they reach the intended tumor site. Such redox-sensitive polymeric micelles deliver paclitaxel directly to the tumor site. In this instance paclitaxel was conjugated to a hydrophilic polymer through a disulfide linkage, forming amphiphilic unimers which assembled into stable micelles. Once within the intracellular environment these linkages are cleaved by reducing agents, such as glutathione, to disrupt the micelle and release the free drug, which elicits a potent cancer-killing effect (Mutlu-Agardan et al., 2020). Nanoparticles that are engineered to target ovarian cancer stem cells could be further enhanced by incorporation of imaging and therapeutic agents. For instance, nanoparticles could be loaded with chemotherapy drugs, such as doxorubicin, and an imaging probe, like gadolinium-DTPA (Gd-DTPA). The utilization of Gd-DTPA as an imaging probe holds the promise of making these nanoparticles visible through MRI (Deng et al., 2022; Margalik et al., 2022). This approach facilitates the tracking of nanoparticle distribution within the body, ensuring the precise location of the tumor.

An effective nano-treatment for ovarian cancer should be able to carry tailored therapeutic agents (such as chemotherapeutic drugs or siRNAs) that are specifically designed based on the patient’s specific ovarian cancer subtype and drug sensitivity (Giordo et al., 2022). The therapeutic agents should be released precisely at the tumor site, potentially with the help of responsive particles, sparing healthy tissues from cytotoxic effects. Incorporating specific targeting ligands to facilitate sub-type specific uptake of identified cancer cells would enable these systems to provide a viable platform for a personalized medicinal strategy (Liang et al., 2023). The detectability of many nanoparticle systems is particularly beneficial to the development of theranostic approaches (Shahriari et al., 2023). To ensure successful clinical translation, pre-clinical studies and clinical trials are necessary to evaluate the safety and efficacy of theranostic nano-systems (Zhang et al., 2022). These multifunctional nano-systems have the potential to revolutionize ovarian cancer management by enabling earlier diagnosis, personalized treatment regimens, and continuous monitoring of therapeutic responses (Farran et al., 2020).

## 5 Challenges and future perspectives

The inter- and intra-patient heterogeneity of ovarian cancers and their associated biocomplexity pose significant challenges to the development of theranostics. These differences are mainly driven by genetic mutations, epigenetic changes, and environmental influences (Corvigno et al., 2016; de Witte et al., 2020). Tumour heterogeneity can make it difficult to accurately diagnose a specific type of cancer. Personalized treatment needs to be implemented and will heavily depend on the availability of sufficiently accurate biomarkers. The ultimate goal will be to predict how different cancer cells will respond to an ever-increasing selection of available pharmaceuticals. Whilst initial treatment might appear successful, the risk of recurrence through resistant cancer cells does remain. Refined theranostic approaches are most suitable to address these problems through accurate diagnosis, tailored treatment, and continued monitoring.

Accelerated and dedicated research into the identification of viable ovarian cancer biomarkers is required to progress clinical implementation. To bring emerging biomarkers into clinical practice, particularly with respect to developing theranostics requires rigorous validation of data from large and diverse patient populations. To achieve this standardization of biomarker testing would be essential to ensure consistent and reliable technologies across different healthcare facilities. The lack of clear regulatory guidelines and safety standards is a major challenge for the development of theranostic agents. To date, only very few theranostic agents have been approved by regulatory agencies such as the FDA (MIRV for instance). The regulatory approval process for theranostic agents is very time-consuming, because they need to be evaluated both for safety and efficacy, as well as diagnostic accuracy (Perera and Morris, 2022). However, to address this new guidance documents and clinical trial designs that are specifically tailored to consider theranostic agents have been developed (Singh et al., 2020).

Despite each of the challenges facing the emergence of new theranostic agents for ovarian cancer, the future of biomarker-driven theranostics hold great potential. Technological advancement and the identification and incorporation of critical biomarkers and targeting factors are likely to address many of these limitations. The integration of multi-omics approaches, which combine data from genomics, transcriptomics, proteomics, and metabolomics to provide a comprehensive view of the disease and reveal novel biomarkers is likely to rapidly advance this field and requires significant research investment (Clifford et al., 2018; Honar et al., 2023).

The integration of real-world data, such as patient outcomes and electronic health records, provides insights beyond clinical trials, revealing how treatments perform in diverse patient populations with comorbidities. Global collaboration among researchers, clinicians, regulators, and industry is essential for the rapid development and implementation of biomarker-driven theranostics. Sharing data, expertise, and resources globally can accelerate progress and improve ovarian cancer patient outcomes. Artificial intelligence (AI) and machine learning approaches and prominent data screening and modelling strategies which have contributed to the advancement of cancer research (Escudero Sanchez et al., 2023). Advanced algorithms have been developed to analyze large datasets, identify subtle patterns and predict patient outcomes with remarkable accuracy (Lu et al., 2020; Liu Y. et al., 2023; Yao et al., 2023; Zhan et al., 2023). These AI-powered models can help clinicians make more informed and personalized treatment decisions. For example, AI can be used to predict the success of immunotherapies based on the expression of extracellular matrix proteins (Geng et al., 2023). Further advancement of AI and machine learning approaches implemented within this field of research could lead to more effective and personalized immunotherapy treatments, amongst other diagnostic, therapeutic and theranostic solutions.

Despite challenges in incorporating emerging biomarkers into clinical practice for ovarian cancer theranostics, ongoing research and technological advancements are converging to enable the development of viable diagnostics, therapeutics, and their combined utility in theranostics. Research guided by the growing knowledge of key biomolecular targets in this field shall not only revolutionize ovarian cancer diagnostics, allowing for earlier critical detection but also develop the field of tumor specific personalised therapies. This is of critical importance to ovarian cancer, particularly due to the heterogenic nature of the disease. Further the advancement of nanomedicine provides a strong basis for the development of highly specialised multimodal particles, which will not only benefit diagnostics and drug delivery but also allow for the combination of these features to develop revolutionary theranostic technologies which are highly versatile and tailorable to personalised conditions. The prospects of this field are bright, and the convergent efforts of researchers, clinicians, regulators, and industry shall rapidly transform the ovarian cancer care landscape in the coming decade.
